# *syn*,*syn*-15,17-Di-2-naphthyl­hexa­cyclo­[10.2.1.1^3,10^.1^5,8^.0^2,11^.0^4,9^]hepta­decane deuterochloro­form monosolvate

**DOI:** 10.1107/S160053680903565X

**Published:** 2009-09-12

**Authors:** Andrew F. DeBlase, Darence Ca Thong, Jocelyn M. Nadeau, David Gebhart, Jonathan Provo, Bruce M. Foxman

**Affiliations:** a Department of Chemistry, Biochemistry and Physics, Marist College, Poughkeepsie, NY 12601, USA; b Olin College, Needham, MA 02492, USA; c Department of Chemistry, MS015, Brandeis University, Waltham, MA 02454, USA

## Abstract

The main molecule of the title compound, C_37_H_36_·CDCl_3_, is a hydro­carbon with two naphthalene segments attached to opposite ends of a rigid norbornylogous spacer with an overall structure that is approximately C-shaped. The dihedral angle between the naphthalene ring planes is 9.27 (7)°. The cleft that exists between the naphthalene rings is large enough that the compound crystallizes with a solvent mol­ecule (CDCl_3_) in the cleft. The CDCl_3_ solvent mol­ecule is present in two disordered orientations in a 3:2 ratio, each involving C—D⋯π to *C*_6_ ring centers.

## Related literature

For examples of related mol­ecules with C-shaped topologies, see: Chou *et al.* (2005 ▶); Klärner *et al.* (2001 ▶); Kurebayashi *et al.* (2001 ▶); Nemoto *et al.* (2000 ▶). For examples of related mol­ecules with the same norbornyl skeleton, see: Bodige *et al.* (1999 ▶); Nadeau *et al.* (2003 ▶). For the Cambridge Structural Database, see: Allen (2002 ▶).
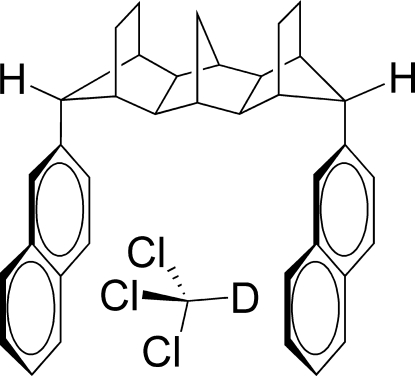


## Experimental

### 

#### Crystal data

C_38_H_37_Cl_3_
*M* *_r_* = 600.07
Triclinic, 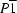

*a* = 6.0833 (4) Å
*b* = 14.6343 (10) Å
*c* = 16.2725 (12) Å
α = 93.641 (5)°
β = 94.437 (4)°
γ = 90.770 (4)°
*V* = 1441.15 (17) Å^3^
*Z* = 2
Mo *K*α radiation
μ = 0.35 mm^−1^
*T* = 120 K
0.59 × 0.29 × 0.24 mm

#### Data collection

Area diffractometer
Absorption correction: multi-scan (*APEX2*; Bruker, 2006 ▶) *T*_min_ = 0.90, *T*_max_ = 0.92
18141 measured reflections
5889 independent reflections
4451 reflections with *I* > 2.0σ(*I*)
*R*_int_ = 0.074

#### Refinement

*R*[*F* ^2^ > 2σ(*F* ^2^)] = 0.058
*wR*(*F* ^2^) = 0.162
*S* = 1.00
5889 reflections
505 parameters
H atoms treated by a mixture of independent and constrained refinement
Δρ_max_ = 0.56 e Å^−3^
Δρ_min_ = −0.83 e Å^−3^

### 

Data collection: *APEX2* (Bruker, 2006 ▶); cell refinement: *APEX2* (Bruker, 2006 ▶); data reduction: *APEX2* (Bruker, 2006 ▶); program(s) used to solve structure: *SIR92* (Altomare *et al.*, 1994 ▶); program(s) used to refine structure: *CRYSTALS* (Betteridge *et al.*, 2003 ▶); molecular graphics: *CAMERON* (Watkin *et al.*, 1996 ▶); software used to prepare material for publication: *CRYSTALS* (Betteridge *et al.*, 2003 ▶).

## Supplementary Material

Crystal structure: contains datablocks global, I. DOI: 10.1107/S160053680903565X/jj2006sup1.cif

Structure factors: contains datablocks I. DOI: 10.1107/S160053680903565X/jj2006Isup2.hkl

Additional supplementary materials:  crystallographic information; 3D view; checkCIF report

## Figures and Tables

**Table 1:** C—D⋯π interactions (Å, °)

C—D⋯*Cg*	C—D	D⋯*Cg*	D⋯*Cg*	*C*—D⋯*Cg*
C38—H381⋯*Cg*1	0.97	2.44	3.399	173
C381—H3811⋯*Cg*2	0.98	2.61	3.521	154

*Cg*1 is the centroid of the C30–C37 ring and

*Cg*2 is the centroid of the C4–C10 ring.

**Fig. 1.**
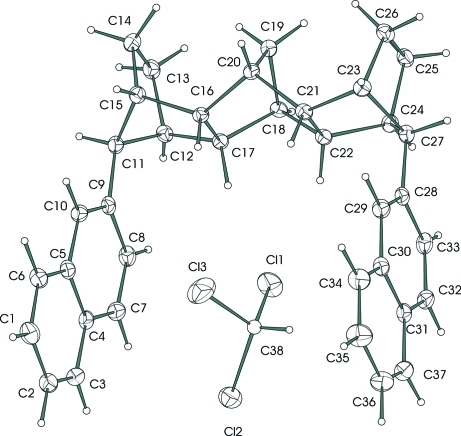
The title compound with displacement ellipsoids drawn at the 50% probability level. H atoms are shown as spheres of arbitary radius. The major component [0.60 (1)] of the disordered CDCl3 solvate is shown.

**Fig. 2.**
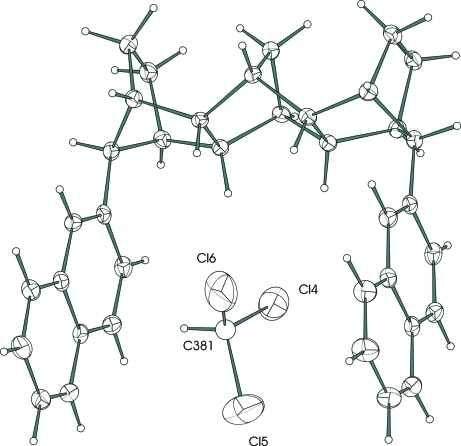
The title compound with displacement ellipsoids drawn at the 50% probability level. H atoms are shown as spheres of arbitary radius. The minor component [0.40 (1)] of the disordered CDCl3 solvate is shown.

**Fig. 3.**
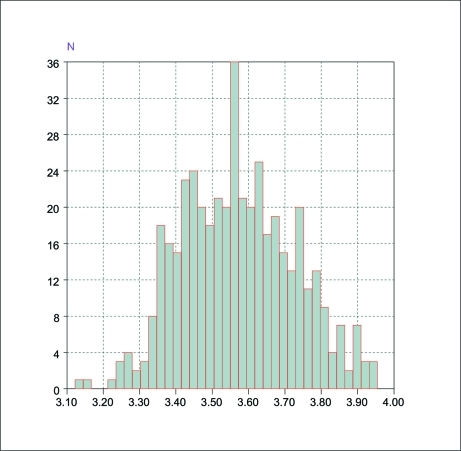
Histogram of C···π distance versus number of structures (378 REFCODES, 443 data points).

**Fig. 4.**
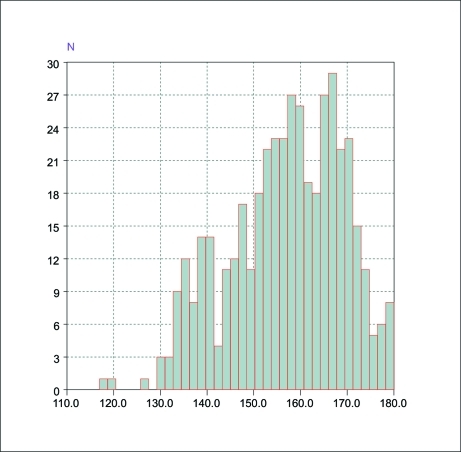
Histogram of C—H···π angle versus number of structures (378 REFCODES, 443 data points).

## supplementary crystallographic information

### Comment

The structure of the title compound, C_37_H_36_^.^CDCl_3_, consists of two naphthalene rings connected to opposite ends of a rigid, norbornylogous hydrocarbon spacer. The overall topology of the molecule is roughly C-shaped, and the cleft that exists between the rings allows for inclusion of a molecule of crystallization, chloroform-*d* (CDCl_3_), as shown in Figs. 1 and 2. Although the naphthalene rings can rotate freely about the C—C bond through which they are attached to the rigid spacer, they are only found in a cofacially stacked, eclipsed conformation. The *anti* conformer is not observed.  The CDCl_3_ within the cleft is in two disordered orientations, occurring in about a 3:2 ratio. Both orientations show interactions with different areas of the naphthalene rings. The most common orientation shows coordination between the chloroform deuterium atom and the π system of the distal ring of the naphthyl group. In this orientation, the C···π and D···π distances are 3.399 and 2.44 Å, repectively, with a C—D···π angle of 172.7°. In the other orientation, the C···π and D···π distances are 3.521 and 2.61 Å, repectively, with a C—D···π angle of 153.9°. To establish the context for the measurements associated with this interaction, the Cambridge Structural Database (Allen, 2002: Version 5.30, including May 2009 updates) was searched for homologous structure-solvent motifs. The search results are organized into two histograms, one for the H(D)···π distance (Fig. 3) and one for the C—H(D)···π angle (Fig. 4). The histograms support the observations, with the values for distance and angle falling well within the range of previously observed values. The observed C···π distance of 3.399 Å was on the low end of recorded distances, whereas the angle of 172.7° was binned at the higher end of the angle range.

### Experimental

Triethylsilane (94 µL; 0.588 mmol) and trifluoroacetic acid (44 µL; 0.588 mmol) were added to a stirring suspension of 15,17-di-2-naphthylhexacyclo[10.2.1.1^3,10^.1^5,8^.0^2,11^.0^4,9^]heptadecane-15,17-diol (25 mg; 0.049 mmol) in CH_2_Cl_2_ under nitrogen. Upon adding the trifluoroacetic acid, the solid gradually went into solution, and the reaction was refluxed for two hours. Upon cooling, solid sodium bicarbonate was added. After 30 minutes, the reaction mixture was filtered and concentrated under reduced pressure. The crude solid was purified by preparative TLC on silica gel (30% CH_2_Cl_2_ in petroleum ether) to give the title compound as the major diastereomer (with evidence of at least one additional diastereomer by NMR) as an off-white solid in 42% yield (10 mg). Single crystals suitable for crystallographic analysis were obtained by slow vapor diffusion of hexanes into a solution of the compound dissolved in chloroform-*d *at room temperature.

### Refinement

The C and Cl atoms were refined by using anisotropic displacement parameters; occupancies of the two disordered CDCl_3_ solvates were constrained to sum to 1.0. The H atoms were located in a difference map, initially refined with soft restraints on the bond lengths and angles to regularize their geometry (C—H in the range 0.93–0.98 and *U*_iso_(H) in the range 1.2–1.5 times *U*_eq_ of the parent atom), after which only their positional parameters were refined (*U*_iso_(H) fixed).

### Crystal data

**Table:**

C_38_H_37_Cl_3_	*Z* = 2
*M**_r_* = 600.07	*F*(000) = 632
Triclinic, *P*1	*D*_x_ = 1.383 Mg m^−^^3^
Hall symbol: -P 1	Mo *K*α radiation, λ = 0.71073 Å
*a* = 6.0833 (4) Å	Cell parameters from 4571 reflections
*b* = 14.6343 (10) Å	θ = 3–26°
*c* = 16.2725 (12) Å	µ = 0.35 mm^−^^1^
α = 93.641 (5)°	*T* = 120 K
β = 94.437 (4)°	Acicular, colourless
γ = 90.770 (4)°	0.59 × 0.29 × 0.24 mm
*V* = 1441.15 (17) Å^3^	

### Data collection

**Table:**

Area diffractometer	5889 independent reflections
Radiation source: fine-focus sealed tube	4451 reflections with *I* > 2.0σ(*I*)
graphite	*R*_int_ = 0.074
φ and ω scans	θ_max_ = 26.4°, θ_min_ = 1.4°
Absorption correction: multi-scan (*APEX2*; Bruker, 2006)	*h* = −7→7
*T*_min_ = 0.90, *T*_max_ = 0.92	*k* = −18→18
18141 measured reflections	*l* = −20→20

### Refinement

**Table:**

Refinement on *F*^2^	Primary atom site location: structure-invariant direct methods
Least-squares matrix: full	Secondary atom site location: difference Fourier map
*R*[*F*^2^ > 2σ(*F*^2^)] = 0.058	Hydrogen site location: inferred from neighbouring sites
*wR*(*F*^2^) = 0.162	H atoms treated by a mixture of independent and constrained refinement
*S* = 1.00	Method = Modified Sheldrick *w* = 1/[σ^2^(*F*^2^) + (0.09*P*)^2^ + 0.62*P*], where *P* = (max(*F*_o_^2^,0) + 2*F*_c_^2^)/3
5889 reflections	(Δ/σ)_max_ = 0.005
505 parameters	Δρ_max_ = 0.56 e Å^−^^3^
0 restraints	Δρ_min_ = −0.83 e Å^−^^3^
0 constraints	

### Special details

**Table:**

Geometry. All e.s.d.'s (except the e.s.d. in the dihedral angle between two l.s. planes) are estimated using the full covariance matrix. The cell e.s.d.'s are taken into account individually in the estimation of e.s.d.'s in distances, angles and torsion angles; correlations between e.s.d.'s in cell parameters are only used when they are defined by crystal symmetry. An approximate (isotropic) treatment of cell e.s.d.'s is used for estimating e.s.d.'s involving l.s. planes.
Refinement. Refinement of *F*^2^ against ALL reflections. The weighted *R*-factor *wR* and goodness of fit *S* are based on *F*^2^, conventional *R*-factors *R* are based on *F*, with *F* set to zero for negative *F*^2^. The threshold expression of *F*^2^ > σ(*F*^2^) is used only for calculating *R*-factors(gt) *etc*. and is not relevant to the choice of reflections for refinement. *R*-factors based on *F*^2^ are statistically about twice as large as those based on *F*, and *R*-factors based on ALL data will be even larger.

### Fractional atomic coordinates and isotropic or equivalent isotropic displacement parameters (Å^2^)

**Table:**

	*x*	*y*	*z*	*U*_iso_*/*U*_eq_	Occ. (<1)
Cl1	0.9320 (6)	0.2872 (2)	0.1765 (2)	0.0336	0.600 (10)
Cl2	1.0165 (7)	0.4616 (3)	0.1051 (3)	0.0301	0.600 (10)
Cl3	0.6094 (4)	0.4332 (3)	0.18090 (16)	0.0440	0.600 (10)
Cl4	0.9649 (15)	0.2877 (5)	0.1738 (5)	0.0836	0.400 (10)
Cl5	0.9765 (16)	0.4486 (6)	0.0912 (6)	0.0735	0.400 (10)
Cl6	0.5802 (11)	0.4017 (5)	0.1684 (4)	0.0928	0.400 (10)
C1	0.7151 (4)	0.66524 (17)	0.32622 (16)	0.0234	
C2	0.9142 (4)	0.67093 (17)	0.28739 (15)	0.0239	
C3	1.0658 (4)	0.60376 (16)	0.29452 (15)	0.0219	
C4	1.0253 (4)	0.52624 (16)	0.33995 (14)	0.0184	
C5	0.8249 (4)	0.52025 (15)	0.37930 (14)	0.0179	
C6	0.6729 (4)	0.59234 (16)	0.37159 (15)	0.0202	
C7	1.1743 (4)	0.45312 (16)	0.34613 (15)	0.0201	
C8	1.1285 (4)	0.37861 (16)	0.38892 (14)	0.0190	
C9	0.9303 (4)	0.37194 (15)	0.42908 (13)	0.0162	
C10	0.7845 (4)	0.44253 (16)	0.42341 (14)	0.0175	
C11	0.8895 (3)	0.29369 (15)	0.48275 (14)	0.0159	
C12	1.0068 (4)	0.20154 (15)	0.46699 (14)	0.0159	
C13	0.9271 (4)	0.14916 (16)	0.53826 (14)	0.0194	
C14	0.6849 (4)	0.18168 (16)	0.54398 (14)	0.0199	
C15	0.6560 (4)	0.24780 (15)	0.47487 (14)	0.0167	
C16	0.6501 (3)	0.20180 (14)	0.38662 (13)	0.0137	
C17	0.8948 (3)	0.16819 (14)	0.38136 (14)	0.0143	
C18	0.8645 (3)	0.06545 (15)	0.35275 (13)	0.0150	
C19	0.6558 (4)	0.03744 (15)	0.39369 (14)	0.0166	
C20	0.5112 (4)	0.11355 (15)	0.35897 (13)	0.0156	
C21	0.5351 (3)	0.10019 (15)	0.26463 (14)	0.0152	
C22	0.7780 (3)	0.06515 (14)	0.26049 (13)	0.0144	
C23	0.3984 (3)	0.02792 (15)	0.20792 (14)	0.0158	
C24	0.7472 (4)	−0.02281 (15)	0.20291 (14)	0.0161	
C25	0.6271 (4)	−0.10214 (16)	0.23961 (15)	0.0181	
C26	0.3867 (4)	−0.06678 (16)	0.24400 (16)	0.0194	
C27	0.5594 (4)	0.00657 (15)	0.14019 (14)	0.0158	
C28	0.6114 (4)	0.08106 (15)	0.08410 (14)	0.0163	
C29	0.4645 (4)	0.14835 (16)	0.06462 (14)	0.0191	
C30	0.5074 (4)	0.21408 (15)	0.00734 (14)	0.0178	
C31	0.7088 (4)	0.21017 (16)	−0.03175 (14)	0.0187	
C32	0.8588 (4)	0.14046 (16)	−0.01134 (15)	0.0196	
C33	0.8119 (4)	0.07889 (16)	0.04453 (14)	0.0190	
C34	0.3567 (4)	0.28382 (17)	−0.01205 (16)	0.0243	
C35	0.4032 (4)	0.34658 (19)	−0.06702 (17)	0.0305	
C36	0.6024 (4)	0.34290 (18)	−0.10577 (16)	0.0293	
C37	0.7516 (4)	0.27660 (17)	−0.08869 (15)	0.0239	
C38	0.8210 (7)	0.3799 (3)	0.1248 (3)	0.0265 (13)*	0.600 (10)
C381	0.8524 (14)	0.3936 (6)	0.1721 (7)	0.048 (3)*	0.400 (10)
H11	0.607 (5)	0.7098 (19)	0.3195 (17)	0.0275*	
H21	0.943 (4)	0.725 (2)	0.2548 (17)	0.0293*	
H31	1.208 (5)	0.6084 (18)	0.2710 (17)	0.0260*	
H61	0.530 (4)	0.5875 (18)	0.4020 (16)	0.0252*	
H71	1.315 (4)	0.4588 (18)	0.3205 (16)	0.0237*	
H81	1.223 (4)	0.3286 (19)	0.3925 (16)	0.0234*	
H101	0.647 (4)	0.4421 (17)	0.4477 (16)	0.0208*	
H111	0.932 (4)	0.3167 (17)	0.5370 (16)	0.0189*	
H121	1.169 (4)	0.2040 (17)	0.4708 (15)	0.0190*	
H131	1.020 (4)	0.1663 (18)	0.5902 (17)	0.0233*	
H132	0.930 (4)	0.0838 (19)	0.5293 (16)	0.0218*	
H141	0.675 (4)	0.2147 (18)	0.5985 (17)	0.0245*	
H142	0.572 (4)	0.1300 (19)	0.5386 (16)	0.0239*	
H151	0.534 (4)	0.2870 (18)	0.4797 (15)	0.0192*	
H161	0.616 (4)	0.2521 (17)	0.3478 (15)	0.0158*	
H171	0.966 (4)	0.2026 (17)	0.3373 (15)	0.0165*	
H181	1.006 (4)	0.0277 (17)	0.3629 (15)	0.0184*	
H191	0.676 (4)	0.0403 (17)	0.4514 (17)	0.0189*	
H192	0.601 (4)	−0.0250 (18)	0.3766 (15)	0.0196*	
H201	0.356 (4)	0.1180 (17)	0.3726 (15)	0.0185*	
H211	0.511 (4)	0.1604 (17)	0.2381 (15)	0.0177*	
H221	0.867 (4)	0.1082 (17)	0.2315 (15)	0.0158*	
H231	0.249 (4)	0.0489 (17)	0.1887 (15)	0.0189*	
H241	0.888 (4)	−0.0443 (17)	0.1796 (15)	0.0187*	
H251	0.703 (4)	−0.1205 (17)	0.2963 (16)	0.0207*	
H252	0.632 (4)	−0.1557 (19)	0.2046 (16)	0.0218*	
H261	0.343 (4)	−0.0617 (18)	0.2997 (17)	0.0229*	
H262	0.279 (4)	−0.1069 (18)	0.2084 (16)	0.0223*	
H271	0.506 (4)	−0.0479 (17)	0.1044 (15)	0.0188*	
H291	0.319 (4)	0.1522 (18)	0.0904 (16)	0.0227*	
H321	0.997 (4)	0.1389 (18)	−0.0381 (16)	0.0234*	
H331	0.915 (4)	0.0338 (18)	0.0586 (16)	0.0222*	
H341	0.228 (5)	0.2868 (19)	0.0184 (17)	0.0297*	
H351	0.308 (5)	0.396 (2)	−0.0806 (18)	0.0354*	
H361	0.633 (5)	0.389 (2)	−0.1449 (18)	0.0351*	
H371	0.891 (5)	0.2712 (19)	−0.1160 (17)	0.0288*	
H381	0.7479	0.3564	0.0729	0.0315*	0.600 (10)
H3811	0.9096	0.4268	0.2240	0.0571*	0.400 (10)

### Atomic displacement parameters (Å^2^)

**Table:**

	*U*^11^	*U*^22^	*U*^33^	*U*^12^	*U*^13^	*U*^23^
Cl1	0.0500 (11)	0.0233 (12)	0.0269 (13)	0.0005 (7)	−0.0086 (8)	0.0115 (9)
Cl2	0.0318 (9)	0.0239 (10)	0.0356 (10)	−0.0007 (7)	0.0087 (7)	0.0022 (7)
Cl3	0.0431 (9)	0.0483 (15)	0.0436 (11)	0.0073 (9)	0.0233 (7)	0.0010 (9)
Cl4	0.155 (6)	0.045 (3)	0.055 (3)	−0.006 (3)	0.039 (3)	0.000 (2)
Cl5	0.102 (5)	0.045 (3)	0.085 (5)	0.035 (3)	0.054 (3)	0.031 (3)
Cl6	0.091 (3)	0.061 (3)	0.130 (4)	−0.003 (2)	0.066 (3)	−0.038 (3)
C1	0.0247 (12)	0.0180 (12)	0.0273 (14)	0.0033 (10)	0.0002 (10)	0.0005 (10)
C2	0.0339 (14)	0.0195 (12)	0.0184 (12)	−0.0035 (10)	0.0031 (10)	0.0007 (10)
C3	0.0247 (12)	0.0218 (12)	0.0194 (12)	−0.0041 (10)	0.0070 (10)	−0.0014 (10)
C4	0.0203 (11)	0.0197 (11)	0.0145 (11)	−0.0040 (9)	0.0014 (8)	−0.0041 (9)
C5	0.0187 (11)	0.0159 (11)	0.0183 (12)	−0.0014 (9)	0.0012 (9)	−0.0037 (9)
C6	0.0190 (11)	0.0181 (11)	0.0230 (12)	−0.0007 (9)	0.0015 (9)	−0.0015 (9)
C7	0.0175 (11)	0.0215 (12)	0.0213 (12)	−0.0020 (9)	0.0046 (9)	−0.0025 (9)
C8	0.0158 (11)	0.0195 (12)	0.0211 (12)	0.0011 (9)	0.0015 (9)	−0.0035 (9)
C9	0.0166 (10)	0.0163 (11)	0.0146 (11)	−0.0022 (8)	−0.0010 (8)	−0.0039 (9)
C10	0.0144 (11)	0.0204 (12)	0.0176 (11)	−0.0028 (9)	0.0039 (9)	−0.0024 (9)
C11	0.0156 (11)	0.0162 (11)	0.0156 (11)	−0.0005 (8)	0.0012 (8)	−0.0018 (9)
C12	0.0134 (11)	0.0156 (11)	0.0185 (12)	−0.0007 (8)	0.0011 (8)	−0.0005 (9)
C13	0.0236 (12)	0.0172 (12)	0.0170 (12)	−0.0005 (9)	−0.0011 (9)	0.0003 (9)
C14	0.0252 (12)	0.0189 (12)	0.0160 (12)	0.0002 (10)	0.0047 (9)	0.0001 (9)
C15	0.0163 (11)	0.0154 (11)	0.0187 (12)	0.0002 (9)	0.0034 (8)	−0.0005 (9)
C16	0.0136 (10)	0.0134 (10)	0.0145 (11)	0.0011 (8)	0.0024 (8)	0.0019 (8)
C17	0.0124 (10)	0.0137 (10)	0.0170 (11)	0.0004 (8)	0.0035 (8)	−0.0001 (8)
C18	0.0134 (10)	0.0148 (11)	0.0170 (11)	−0.0005 (8)	0.0012 (8)	0.0020 (9)
C19	0.0190 (11)	0.0157 (11)	0.0148 (11)	−0.0019 (9)	0.0011 (9)	−0.0001 (9)
C20	0.0134 (10)	0.0170 (11)	0.0165 (11)	−0.0009 (8)	0.0041 (8)	−0.0014 (9)
C21	0.0125 (10)	0.0137 (11)	0.0196 (12)	0.0005 (8)	0.0029 (8)	0.0007 (9)
C22	0.0124 (10)	0.0131 (10)	0.0180 (11)	0.0008 (8)	0.0029 (8)	0.0008 (9)
C23	0.0112 (10)	0.0189 (11)	0.0173 (11)	0.0003 (8)	0.0010 (8)	0.0008 (9)
C24	0.0161 (11)	0.0156 (11)	0.0167 (11)	0.0017 (8)	0.0018 (8)	0.0004 (9)
C25	0.0192 (11)	0.0133 (11)	0.0219 (12)	0.0000 (9)	0.0029 (9)	−0.0013 (9)
C26	0.0169 (11)	0.0179 (11)	0.0229 (12)	−0.0040 (9)	0.0017 (9)	−0.0017 (9)
C27	0.0146 (10)	0.0152 (11)	0.0171 (11)	0.0004 (8)	0.0010 (8)	−0.0019 (9)
C28	0.0163 (11)	0.0163 (11)	0.0157 (11)	−0.0026 (8)	0.0007 (8)	−0.0040 (9)
C29	0.0165 (11)	0.0197 (12)	0.0208 (12)	−0.0002 (9)	0.0030 (9)	−0.0019 (9)
C30	0.0179 (11)	0.0167 (11)	0.0181 (11)	−0.0007 (9)	0.0005 (9)	−0.0026 (9)
C31	0.0201 (11)	0.0188 (11)	0.0164 (11)	−0.0033 (9)	0.0012 (9)	−0.0048 (9)
C32	0.0164 (11)	0.0212 (12)	0.0211 (12)	−0.0008 (9)	0.0054 (9)	−0.0042 (9)
C33	0.0163 (11)	0.0200 (12)	0.0202 (12)	0.0035 (9)	0.0012 (9)	−0.0038 (9)
C34	0.0221 (12)	0.0240 (13)	0.0274 (14)	0.0030 (10)	0.0026 (10)	0.0034 (10)
C35	0.0303 (14)	0.0263 (14)	0.0359 (16)	0.0060 (11)	0.0028 (11)	0.0094 (12)
C36	0.0386 (15)	0.0241 (13)	0.0262 (14)	−0.0012 (11)	0.0055 (11)	0.0071 (11)
C37	0.0252 (13)	0.0260 (13)	0.0207 (12)	−0.0037 (10)	0.0056 (10)	−0.0019 (10)

### Geometric parameters (Å, °)

**Table:**

Cl1—C38	1.756 (5)	C18—C22	1.552 (3)
Cl2—C38	1.736 (6)	C18—H181	1.04 (3)
Cl3—C38	1.794 (6)	C19—C20	1.537 (3)
Cl4—C381	1.704 (12)	C19—H191	0.94 (3)
Cl5—C381	1.795 (12)	C19—H192	0.98 (3)
Cl6—C381	1.658 (11)	C20—C21	1.553 (3)
C1—C2	1.413 (3)	C20—H201	0.99 (2)
C1—C6	1.368 (3)	C21—C22	1.575 (3)
C1—H11	0.94 (3)	C21—C23	1.548 (3)
C2—C3	1.361 (4)	C21—H211	1.01 (3)
C2—H21	1.00 (3)	C22—C24	1.544 (3)
C3—C4	1.422 (3)	C22—H221	0.99 (2)
C3—H31	0.98 (3)	C23—C26	1.542 (3)
C4—C5	1.425 (3)	C23—C27	1.550 (3)
C4—C7	1.415 (3)	C23—H231	0.99 (3)
C5—C6	1.417 (3)	C24—C25	1.540 (3)
C5—C10	1.412 (3)	C24—C27	1.556 (3)
C6—H61	1.04 (3)	C24—H241	1.01 (3)
C7—C8	1.367 (3)	C25—C26	1.563 (3)
C7—H71	0.98 (3)	C25—H251	1.05 (3)
C8—C9	1.421 (3)	C25—H252	0.94 (3)
C8—H81	0.94 (3)	C26—H261	0.96 (3)
C9—C10	1.373 (3)	C26—H262	1.00 (3)
C9—C11	1.513 (3)	C27—C28	1.511 (3)
C10—H101	0.95 (3)	C27—H271	0.99 (3)
C11—C12	1.550 (3)	C28—C29	1.374 (3)
C11—C15	1.556 (3)	C28—C33	1.423 (3)
C11—H111	0.94 (3)	C29—C30	1.417 (3)
C12—C13	1.535 (3)	C29—H291	1.01 (3)
C12—C17	1.550 (3)	C30—C31	1.424 (3)
C12—H121	0.99 (3)	C30—C34	1.415 (3)
C13—C14	1.562 (3)	C31—C32	1.416 (3)
C13—H131	1.00 (3)	C31—C37	1.420 (3)
C13—H132	0.96 (3)	C32—C33	1.363 (3)
C14—C15	1.531 (3)	C32—H321	0.98 (3)
C14—H141	0.99 (3)	C33—H331	0.94 (3)
C14—H142	1.01 (3)	C34—C35	1.364 (4)
C15—C16	1.545 (3)	C34—H341	0.96 (3)
C15—H151	0.95 (3)	C35—C36	1.408 (4)
C16—C17	1.580 (3)	C35—H351	0.95 (3)
C16—C20	1.556 (3)	C36—C37	1.365 (4)
C16—H161	1.01 (2)	C36—H361	0.98 (3)
C17—C18	1.550 (3)	C37—H371	0.99 (3)
C17—H171	1.02 (2)	C38—H381	0.965
C18—C19	1.541 (3)	C381—H3811	0.984
			
C2—C1—C6	120.4 (2)	C19—C20—H201	119.6 (14)
C2—C1—H11	120.8 (17)	C16—C20—H201	113.0 (14)
C6—C1—H11	118.8 (17)	C21—C20—H201	113.0 (14)
C1—C2—C3	120.3 (2)	C20—C21—C22	102.95 (17)
C1—C2—H21	119.3 (16)	C20—C21—C23	122.72 (18)
C3—C2—H21	120.4 (16)	C22—C21—C23	102.85 (17)
C2—C3—C4	120.7 (2)	C20—C21—H211	109.9 (14)
C2—C3—H31	121.5 (16)	C22—C21—H211	112.7 (14)
C4—C3—H31	117.7 (16)	C23—C21—H211	105.6 (14)
C3—C4—C5	119.1 (2)	C18—C22—C21	103.20 (17)
C3—C4—C7	122.6 (2)	C18—C22—C24	123.41 (18)
C5—C4—C7	118.3 (2)	C21—C22—C24	102.85 (16)
C4—C5—C6	118.4 (2)	C18—C22—H221	110.2 (14)
C4—C5—C10	118.8 (2)	C21—C22—H221	110.3 (14)
C6—C5—C10	122.7 (2)	C24—C22—H221	106.3 (14)
C5—C6—C1	121.0 (2)	C21—C23—C26	113.65 (18)
C5—C6—H61	117.2 (15)	C21—C23—C27	100.81 (16)
C1—C6—H61	121.8 (15)	C26—C23—C27	99.68 (18)
C4—C7—C8	121.2 (2)	C21—C23—H231	113.8 (14)
C4—C7—H71	117.8 (15)	C26—C23—H231	111.4 (14)
C8—C7—H71	121.0 (15)	C27—C23—H231	116.4 (15)
C7—C8—C9	121.3 (2)	C22—C24—C25	114.65 (19)
C7—C8—H81	122.5 (16)	C22—C24—C27	100.62 (17)
C9—C8—H81	116.2 (16)	C25—C24—C27	99.23 (17)
C8—C9—C10	118.0 (2)	C22—C24—H241	113.5 (14)
C8—C9—C11	121.1 (2)	C25—C24—H241	110.9 (14)
C10—C9—C11	120.7 (2)	C27—C24—H241	116.9 (14)
C5—C10—C9	122.4 (2)	C24—C25—C26	103.21 (18)
C5—C10—H101	114.9 (16)	C24—C25—H251	113.2 (14)
C9—C10—H101	122.6 (16)	C26—C25—H251	114.7 (14)
C9—C11—C12	119.44 (18)	C24—C25—H252	110.0 (16)
C9—C11—C15	118.04 (18)	C26—C25—H252	111.8 (16)
C12—C11—C15	93.27 (16)	H251—C25—H252	104 (2)
C9—C11—H111	105.1 (15)	C23—C26—C25	103.04 (17)
C12—C11—H111	108.2 (16)	C23—C26—H261	111.2 (16)
C15—C11—H111	112.6 (15)	C25—C26—H261	112.2 (15)
C11—C12—C13	99.73 (18)	C23—C26—H262	109.1 (15)
C11—C12—C17	100.98 (17)	C25—C26—H262	111.3 (15)
C13—C12—C17	113.39 (17)	H261—C26—H262	110 (2)
C11—C12—H121	116.4 (15)	C23—C27—C24	93.55 (17)
C13—C12—H121	109.7 (14)	C23—C27—C28	118.46 (19)
C17—C12—H121	115.4 (14)	C24—C27—C28	117.74 (18)
C12—C13—C14	103.27 (18)	C23—C27—H271	110.1 (14)
C12—C13—H131	110.0 (15)	C24—C27—H271	109.0 (14)
C14—C13—H131	111.4 (15)	C28—C27—H271	107.3 (14)
C12—C13—H132	114.3 (15)	C27—C28—C29	122.4 (2)
C14—C13—H132	110.5 (15)	C27—C28—C33	119.7 (2)
H131—C13—H132	107 (2)	C29—C28—C33	117.7 (2)
C13—C14—C15	102.96 (18)	C28—C29—C30	122.2 (2)
C13—C14—H141	108.2 (15)	C28—C29—H291	120.7 (15)
C15—C14—H141	110.5 (15)	C30—C29—H291	117.1 (15)
C13—C14—H142	113.8 (15)	C29—C30—C31	119.0 (2)
C15—C14—H142	113.4 (15)	C29—C30—C34	122.1 (2)
H141—C14—H142	108 (2)	C31—C30—C34	118.9 (2)
C14—C15—C11	99.77 (18)	C30—C31—C32	118.3 (2)
C14—C15—C16	114.65 (18)	C30—C31—C37	118.8 (2)
C11—C15—C16	100.76 (17)	C32—C31—C37	122.9 (2)
C14—C15—H151	113.5 (15)	C31—C32—C33	120.9 (2)
C11—C15—H151	117.2 (15)	C31—C32—H321	117.5 (15)
C16—C15—H151	110.2 (15)	C33—C32—H321	121.6 (15)
C15—C16—C17	102.56 (16)	C28—C33—C32	121.9 (2)
C15—C16—C20	122.89 (18)	C28—C33—H331	118.2 (16)
C17—C16—C20	102.65 (17)	C32—C33—H331	119.9 (16)
C15—C16—H161	106.0 (14)	C30—C34—C35	120.8 (2)
C17—C16—H161	111.2 (13)	C30—C34—H341	116.3 (17)
C20—C16—H161	111.0 (13)	C35—C34—H341	122.7 (17)
C12—C17—C16	102.74 (17)	C34—C35—C36	120.5 (2)
C12—C17—C18	122.80 (18)	C34—C35—H351	123.4 (18)
C16—C17—C18	103.31 (16)	C36—C35—H351	116.1 (18)
C12—C17—H171	108.3 (13)	C35—C36—C37	120.4 (2)
C16—C17—H171	108.7 (13)	C35—C36—H361	118.9 (17)
C18—C17—H171	110.0 (14)	C37—C36—H361	120.6 (17)
C17—C18—C19	102.78 (17)	C31—C37—C36	120.6 (2)
C17—C18—C22	104.64 (17)	C31—C37—H371	117.0 (16)
C19—C18—C22	101.88 (17)	C36—C37—H371	122.3 (16)
C17—C18—H181	113.6 (14)	Cl3—C38—Cl1	110.8 (3)
C19—C18—H181	118.4 (14)	Cl3—C38—Cl2	109.7 (3)
C22—C18—H181	113.8 (14)	Cl1—C38—Cl2	113.8 (3)
C18—C19—C20	95.48 (17)	Cl3—C38—H381	105.4
C18—C19—H191	112.9 (15)	Cl1—C38—H381	108.3
C20—C19—H191	114.2 (15)	Cl2—C38—H381	108.5
C18—C19—H192	114.0 (14)	Cl5—C381—Cl4	105.6 (7)
C20—C19—H192	114.2 (14)	Cl5—C381—Cl6	114.0 (7)
H191—C19—H192	106 (2)	Cl4—C381—Cl6	118.6 (6)
C19—C20—C16	102.68 (17)	Cl5—C381—H3811	105.9
C19—C20—C21	102.59 (17)	Cl4—C381—H3811	105.6
C16—C20—C21	104.22 (17)	Cl6—C381—H3811	106.2

### Hydrogen-bond geometry (Å, °)

**Table:**

*D*—H···*A*	*D*—H	H···*A*	*D*···*A*	*D*—H···*A*
C38—H381···Cg1	0.97	2.44	3.399	173
C381—H3811···Cg2	0.98	2.61	3.521	154
